# Antibacterial efficiency over time and barrier properties of wood coatings with colloidal silver

**DOI:** 10.1007/s00253-023-12710-1

**Published:** 2023-08-08

**Authors:** Massimo Calovi, Valentina Coroneo, Stefano Rossi

**Affiliations:** 1 Department of Industrial Engineering, University of Trento, Via Sommarive 9, 38123 Trento, Italy; 2 Department of Medical Sciences and Public Health, University of Cagliari, S.P.8 Monserrato, 09042 Cagliari, Italy

**Keywords:** Colloidal silver, Wood paint, Antimicrobial activity, Time effectiveness, Coatings durability

## Abstract

**Abstract:**

This work aims to represent a standard application for understanding the extent of the antibacterial efficacy of coatings with different amounts of colloidal silver on wooden surfaces over time. The key variable that was intended to be evaluated in this study was the “time efficiency,” with concerns about the possible efficacy in the durability of the surfaces. By highlighting the “expiry date” of the agents, as in the case with other products, the study aimed to confirm the validity of the simulation tests conducted in the laboratory with degradation tests. Furthermore, the role of the silver amount on the barrier performance of the coatings was assessed by liquid resistance, water uptake, and perspiration tests, evaluating the aesthetic durability of the coatings by means of colorimetric analyses. Ultimately, this work demonstrates that these coatings may represent alternatives in terms of prolonged antimicrobial activity when compared with the biocide agents currently in use, capable to offer good resistance to detergent solutions and to water. Nevertheless, due to silver’s susceptibility to extended exposure to acidic solutions, the findings of the research discourage the utilization of colloidal silver in wood paints intended for use in public settings.

**Key points:**

• *Colloidal silver does not alter the deposition process and does not introduce defects in the wood paint.*

• *Coatings containing silver show high antimicrobial activity over time, against both E.coli and S.aureus.*

• *The silver-based filler resists contact with detergents and aqueous solutions but suffers oxidation processes in acidic environments.*

**Supplementary Information:**

The online version contains supplementary material available at 10.1007/s00253-023-12710-1.

## Introduction

Human exposure to biological agents such as air, contact, cross-contamination, and the assessment of their airborne or sedimented microbiological concentration on surfaces are currently the subjects of study in Public Health, considering the recent COVID-19 pandemic. Indeed, these mechanisms of exposure represent the semi-direct and direct transmission of numerous pathogenic microorganisms (Pontello and Auxilia 2022). The findings from microbiological surveillance carried out on surfaces, with the objective of evaluating the efficacy of sanitation protocols, revealed that the presence of contaminating microorganisms is frequently unevenly distributed. This argument is based on the presence of the biocides used, which require more representative techniques for sampling and new antibacterial formulations (Jones et al. 2020). The phenomenon of antibiotic resistance by bacteria is increasing worldwide and is a major concern of the World Health Organization (WHO). The root cause of the issue is linked not only to the production and processing of animal-based food but also to healthcare procedures, specifically the use of biocides for disinfecting environments, equipment, and surfaces. This significantly contributes to the emergence of antibiotic-resistant bacterial strains (Ghosh et al. 2021).

One of the most popular methods for making antibacterial surfaces is the implementation of protective coatings with silver, which offers high antimicrobial activity (Mohamed et al. 2020; Sandle 2012). Indeed, the production of wood coatings containing silver showed antibacterial properties against Gram-negative bacteria (represented by *Escherichia coli*) and Gram-positive bacteria (represented by *Staphylococcus aureus*), demonstrating good antimicrobial activity (*R*) values based on the protocol BS ISO 22196: 2011 (BS-ISO-22196 2011). The application of such coatings protects the wood while minimizing the risk of being a vehicle for the chain of transmission of infectious diseases. However, this practice is not widely adopted at present because wood usage is not typically associated with critical or semi-critical environments.

It can be argued that health can be defined as “the state of the general physical and mental well-being of individuals” and not anymore as “the condition of no longer as the absence of diseases” as pieces of evidence show how indoor health conditions have improved over time (Leonardi 2018). Indeed, the positive impact on individual well-being and the effectiveness of antimicrobial activities could contribute to reducing the risk of exposure to microorganisms by promoting a healthier environment. This could be implemented with the introduction of inherent protective features in the surfaces of everyday environments. Therefore, indoor settings like schools, meeting rooms, and doctor’s offices (including desks) are marked by crowded conditions, making them potential areas where the practical implementation of this research could help reduce the risk of infections. From this point of view, the presence of so-called “warm” materials, such as those made of wood, is not negligible in the healthcare-related environment.

Although there is growing interest in developing antibacterial coatings for wood and assessing their antimicrobial effectiveness, there is a lack of research in the literature regarding the long-term antimicrobial efficiency of these surfaces. In the context of the current emergence of antibiotic-resistant bacterial strains, silver represents a valid and functional solution, but it is necessary to evaluate its effectiveness over time. Therefore, this work heavily highlights this aspect, analyzing the evolution of the antibacterial performance of wood coatings over a long period of time.

Moreover, although wood is recognized for its unique appearance, the industry frequently needs for wood paints with distinctive aesthetic qualities and eye-catching hues that can offer the wooden product novel appearances (Wiemann 2010). Consequently, colloidal silver, which can provide vivid orange-red coloring in transparent paints at low concentrations (Calovi et al. 2023), could be exploited as an alternative antibacterial pigment.

Thus, in this paper the antibacterial efficacy of coatings with colloidal silver in the long-term of wooden surfaces was evaluated through the application of an experimental protocol. The aim was to understand the extent of their protection considering “the time variable” as the main effect to be investigated along with the effectiveness in the time duration by highlighting the “expiry date” as in the case with other products. Furthermore, the effect of silver in influencing the morphology of the coatings and their barrier performance was evaluated through specific tests for wood paints.

## Materials and methods

### Materials

The 50 × 50 × 2 mm^3^ poplar wood panels were purchased from OBI GmbH & Co (Wermelskirchen, Germany). The colloidal silver source (65–75 wt.% Ag) was purchased from Sigma-Aldrich (St. Louis, MO, USA) and used as received. The transparent water-borne paint WTVE097E30 supplied by ICRO Coatings S.p.A. (Bergamo, Italy) is based on polyurethane, di(propylen glycol) monomethyl ether, paraffin and hydrocarbon waxes, di(propylenglicol) n-butyl ether, and 2-butoxyethanol. As it possesses a water concentration ≥ 75% and no solvents, the paint is defined as a water-based product with a dry residue equal to 34% and a specific weight of 1.05 g/cm^3^. The commercial detergent disinfectant product Suma Bac D10 Cleaner and Sanitizer (Diversey-Fort Mill, SC, USA), containing benzalkonium chloride (3.0–10.0 wt.%), and the cataphoretic red ink Catafor 502XC (Arsonsisi, Milan, Italy) were purchased and used for the liquid resistance tests. Sodium chloride and lactic acid were purchased from Sigma-Aldrich and used for the perspiration resistance test.

Nutrient broth (NB), nutrient agar (NA), plate counting agar (PCA), and phosphate buffered saline (1 × PBS) were bought from Microbiol S.n.c. (Uta, Cagliari, Italy) and have been used for bacterial cultures and antibacterial tests. PBS was used for serial dilutions and to recover bacterial cells from test samples. *Staphylococcus aureus* ATCC 6538 and *Escherichia coli* ATCC 25922 were bought from TCS Biosciences and Microbiology (Bruker) which operates along the regulation ISO 17034 to produce reference materials. All the materials used in this study can be found in the Supplementary Materials (Table S1).

### Samples production

The samples were produced following the procedure described in the previous study (Calovi et al. 2023). To achieve a smooth finish, the wood panels were first polished employing a 320 grit sandpaper. The pre-treated hardwood surfaces were then painted with the water-borne paint supplied by ICRO, and the films were left to drying for 5 h at room temperature. The aforementioned procedure was carried out twice. Nevertheless, colloidal silver was mixed with the paint used for the second application. Hence, the coatings were made up of two distinct layers, with specific purpose. The first, free of silver, was deposited to ensure enough protection to the wooden substrate, while the second outermost layer was applied to deliver the coating with effective antibacterial features provided by the colloidal silver. Prior to applying the second layer, the colloidal silver granules were first partially dissolved in water to become substances smaller than µm, forming a homogeneous suspension. The silver-based commercial product used is defined as “colloidal” as this suspension, unlike typical solutions containing nanopowders, represents a heterogeneous system, which appears turbid. The dissolution process was accomplished by mixing the solution for 30 min with an ultrasonic probe. Thus, the paint was diluted to 90% employing the aqueous silver-based solution. Two different samples were produced, employing two specific amounts of colloidal silver in the formulation of the silver-based aqueous suspensions. Ten milliliters of water were mixed with 0.1 g and 0.5 g of colloidal silver, respectively. Consequently, the two colloidal silver suspensions were added separately to two batches of 90 ml of water-borne paint, to obtain two final products of 100 g containing 0.1 wt.% and 0.5 wt.% of silver, respectively. A sample made of pure polyurethane layer, free of silver filler, was created as a comparison reference for the performance of the two samples with silver additives, in order to evaluate the behavior and impact of the antibacterial filler. Table 1 summarizes the three samples series formulation, with the corresponding nomenclature.

**Table 1:** Samples nomenclature

Samples nomenclature	Colloidal silver concentration (wt.%)	
	First layer	Second layer
Ag0	/	/
Ag01	/	0.1
Ag05	/	0.5

### Coatings morphology

The colloidal silver powder was analyzed by means of low vacuum scanning electron microscope SEM JEOL IT 300 (JEOL, Akishima, Tokyo, Japan) observations. In order to determine if the addition of colloidal silver has caused morphology issues in the polyurethane matrix, the cross section of the coatings was examined using the optical stereomicroscope Nikon SMZ25 (Nikon Instruments, Amstelveen, Netherlands). These analyses were also carried out to measure the thickness of the three series of coatings and to evaluate the dispersion of silver within the polymeric matrix. In order to evaluate the aesthetic stability of the coatings, the samples were subjected to colorimetric measurements 18 months after their production. The measurements were performed with of a Konica Minolta CM-2600d spectrophotometer (Konica Minolta, Tokyo, Japan) with a D65/10° illuminant/observer configuration in SCI mode. The test was performed on 5 samples per series, while 10 colorimetric analyses were carried out for each sample.

### Coatings antibacterial efficacy

The experiments were conducted at the Hygiene Laboratory of the University of Cagliari, accredited UNI EN ISO/IEC 17025:2018 (general requirements for the competence of testing and calibration laboratories). The protective efficacy over time of wood surfaces containing coatings with colloidal silver was evaluated by applying the protocol BS ISO 22196: 2011 (BS-ISO-22196 2011) at different times. Antibacterial activity (*R*) was assessed at the time of production of the silver-containing coatings (T0) and then repeated at the midpoint of the product’s shelf-life estimated at 6 months (T1), at the end of the shelf-life, corresponding to 12 months (T2) and finally at an additional 50% of the assumed shelf-life corresponding to 18 months (T3). Each time (T0, T1, T2, T3), tests were conducted using the untreated specimens made from wood with water-borne paint (Ag0) and treated specimens Ag01 and Ag05.

All specimens were disinfected by immersion in chlorhexidine digluconate (0.02%) and benzalkonium chloride (0.2%) for 10 min, washed with sterile water, and subsequent ultraviolet (UV) radiation for 1 h per side in a sterile tray within a laminar flow hood (Kampf et al. 2020; Martí et al. 2018).

*Staphylococcus a*ureus ATCC 6538 and *Escherichia coli* ATCC 25922 (class II) were used for the evaluation of antibacterial activity against Gram-positives and Gram-negatives, respectively. Microbial suspensions were prepared from lyophilised pellets (according to EN1276) resuspended in a Nutrient Broth medium, and the concentration was determined by spectrophotometric reading (Agilent Technologies Cary 60). The bacteria were pre-cultured overnight at 35 ± 1 °C on NA plates and then inoculated in 10 ml of NB 1/500 to an initial bacterial concentration of 1.5 × 10^8^ cells/mL (McFarland standard 0, 5). This suspension was then diluted in sterile PBS 1 × to obtain a suspension (inoculum) with an estimated bacterial concentration between 1.0 × 10^6^ and 6.0 × 10^6^ cells/mL, with a target concentration of 2.5 × 10^6^ cells/mL. CFU/ml colony-forming units were determined by tenfold dilution and plating on nutrient agar (NA) plates followed by overnight incubation at 35 ± 1 °C.

The inoculum (100 µL) was applied to the three sets of highly disinfected surfaces (2.5 cm × 2.5 cm) and coated with polypropylene film (2.0 cm × 2.0 cm). The three replicates of the samples Ag0/T24h, Ag01/T24h, and Ag05/T24h were incubated at a temperature of 35 ± 1 C for 24 h at 90% relative humidity. The control sample Ag0/T0, representing inoculation at T0, was processed immediately after direct contact with the bacteria. The bacterial cells were recovered from Ag0/T0 by washing with 10 ml of sterile PBS 1 × , vortexing for 1 min to detach them, and then serial dilutions were included and incubated in a PCA plate at 35 ± 1 °C for 24 h for colony counting. After the incubation period of 24 h (T24h), the bacterial cells were recovered from the test samples (Ag01, Ag05) and from the untreated sample (Ag0) by adding 10 ml of 1 × sterile PBS and vortexing for 1 min.

After this last step, a tenfold dilution in PBS 1 × was performed, and 1 ml of each sample was included in the PCA plates and incubated at 35 °C for 24 and 48 h to enumerate viable bacteria. Bacterial colonies were counted according to BS ISO 22196 protocol and expressed as:

- *N* (cells/cm^2^) = (100 C D V)/A, where *N* is the number of viable bacteria recovered per cm^2^ for the sample,
- *C*, the number of colony-forming units (CFU) determined by counting on agar plates for each group of samples,
- *D*, the dilution factor for the counted plates,
- *V*, the volume in ml of the solution to recover bacteria from the samples
- *A*, the surface area of the cover film in mm^2^.

The antibacterial activity of the treated surfaces was calculated using the following equation:

$$R=\left({U}_{t}-{U}_{0}\right)-\left({A}_{t}- {U}_{0}\right)= {U}_{t}- {A}_{t},$$

where *U*_0_ = log10 mean of the number of viable bacteria recovered from the untreated surface immediately after inoculation; *U*_t_ = log10 mean of the number of viable bacteria recovered; *A*_t_ = log10 mean of the number of viable bacteria recovered from the treated sample after 24 h of incubation.

### Coatings liquid resistance

The chemical resistance test was carried out in accordance with the GB/T 1733–93 standard (GB/T1733-93 1993), to determine the possible influence of the silver additive on the polyurethane matrix's barrier performance. A filter paper was soaked in a 15% sodium chloride solution, 70% ethanol, detergent, and red ink, respectively. Thus, the filter papers were positioned on top of the coatings, and a glass cover was placed over it. The glass covers and filter papers were taken off after 24 h, and the residual liquid on the coated surface was absorbed. The colorimetric investigations were used to analyze the imprint and discoloration. These analyses were performed with a Konica Minolta CM-2600d spectrophotometer (Konica Minolta, Tokyo, Japan) with a D65/10° illuminant/observer configuration in SCI mode. The test was performed on 5 samples per series, while 10 colorimetric analyses were carried out for each sample. Moreover, in accordance with EN 927–5:2007 standard (EN927-5 2007), the real barrier capabilities of the paints against water absorption were evaluated using the liquid water uptake test. To prevent water uptake occurrences by the wooden substrate, silicone was used to thoroughly cover the 5 uncoated sides of the 40 × 40 × 2 mm^3^ poplar wood panels. The samples were preconditioned at 20 °C and 65% RH before being allowed to float in a water-filled container. To calculate the moisture uptake, expressed as g/m^2^, the mass gain was measured at 0, 6, 24, 48, 72, and 96 h. The test was performed on 5 samples per series. Moreover, since the wood paint is designed to exhibit good durability, the samples were subjected to a specific accelerated degradation test, following the ISO 12870:2016 standard (Sect. 8.5) (ISO12870 2016), to evaluate their perspiration resistance. The samples were exposed to the aggressive solution that replicated human sweat for a total of 24 h at temperature of 60 °C, analyzing the surface decay after 8 h and at the conclusion of the test. With this characterization method, the component experienced a simulation of repeated interaction with human skin. The same procedure was applied employing simple demineralized water instead of the sweat-simulating solution, to reproduce the exposure of the sample to repeated washing or to external agents. Once again, the behavior of the samples was analyzed by colorimetric measurements, while the evolution of the silver amount in the coatings was evaluated with energy-dispersive X-ray spectroscopy (EDXS, Bruker, Billerica, MA, USA) analyses. The test was performed on 5 samples per series, and 10 colorimetric analyses were carried out for each sample.

## Results

### Coatings morphology

**Fig. 1:**
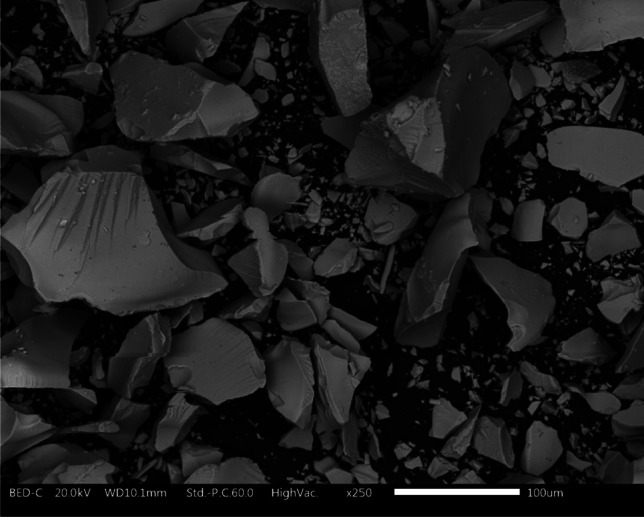
Colloidal silver granules acquired by SEM

Figure 1 reveals the appearance of the commercial colloidal silver product. It displays as little, gray particles with a size range of a few µm to over 200 µm. Prior to being added to the water-borne paint, the granules were fully dissolved in an aqueous solution, indicating the great solubility of this product in water as shown by recent research (Calovi et al. 2023; Calovi and Rossi 2022).

**Fig. 2:**
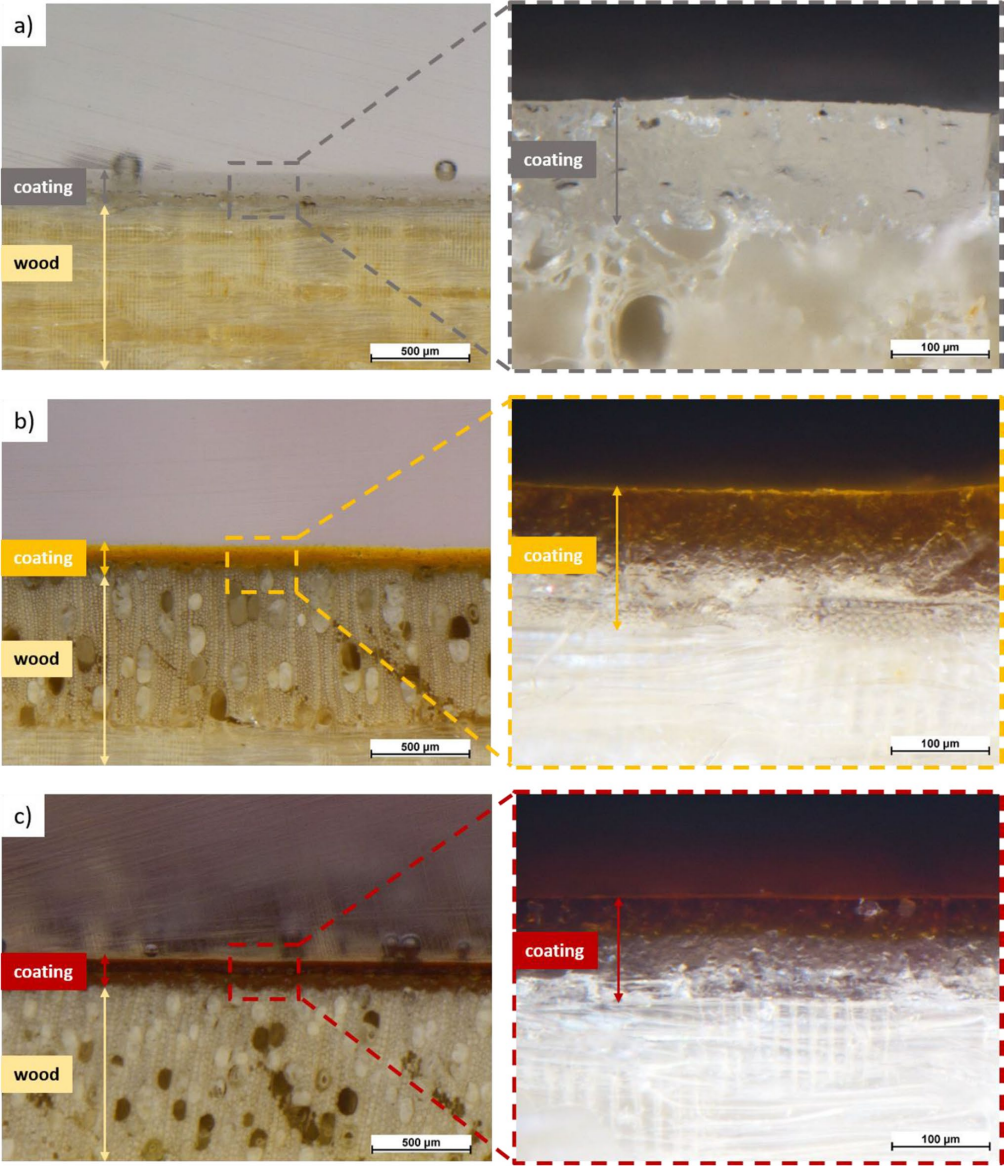
Optical microscope micrographs of the cross-section of sample Ag0 (**a**), sample Ag01 (**b**), and sample Ag05 (**c**)

As was reported in detail in the previous study (Calovi et al. 2023), the color of the aqueous solution varies towards orange hues, depending on the amount of silver granules that has been dissolved in. As a result, the aspect of the clear polyurethane-based paint drastically changes after being diluted with the Ag-aqueous solution. Consequently, the three series of coatings possess quite distinct appearances, as demonstrated by the images in Fig. 2, which were acquired with the optical stereomicroscope. The cross-sectional images of the coatings of samples Ag01 and Ag05, in Figs. 2b and c, respectively, allow to appreciate the different color between the first transparent layer and the film subsequently applied with the solution containing silver. The three series of coatings possess comparable thickness, of about 120 µm, as both depositions produced a layer of 60 µm each. Furthermore, the coatings appear compact and free from defects and bubbles, with a homogeneous and intense color.

**Fig. 3:**
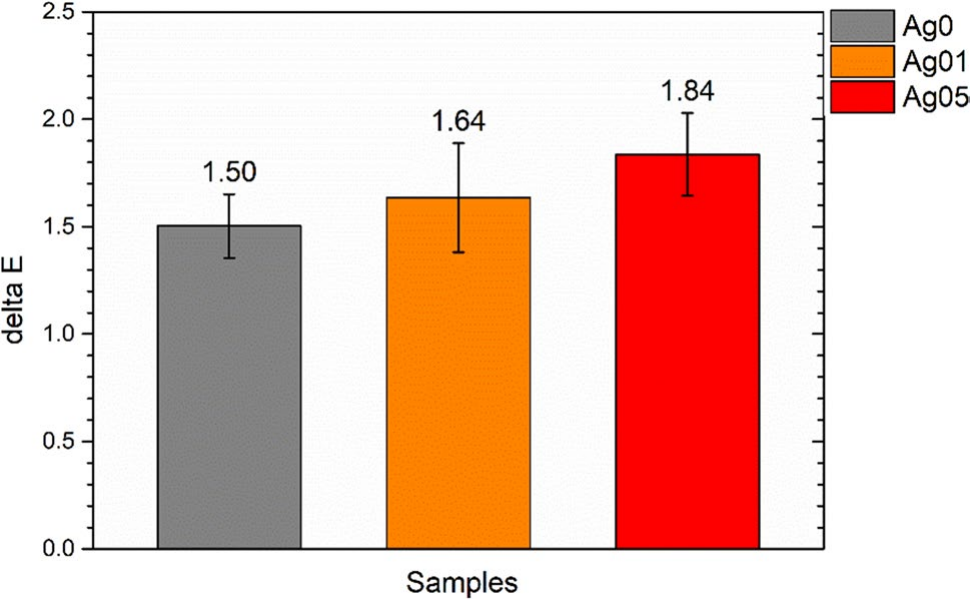
Coatings color changes after 18 months

The aesthetic stability of the coatings was evaluated by colorimetric measurements performed 18 months after their production. Figure 3 shows the color change Δ*E* of the three series of coatings, calculated according to the ASTM E308-18 standard (ASTM-E308-18 2018):

$$\Delta E=[{{\left({\Delta L}^{*}\right)}^{2}+{\left({\Delta a}^{*}\right)}^{2}+{\left({\Delta b}^{*}\right)}^{2}]}^{1/2 },$$

where the colorimetric coordinates L*, a*, and b* correspond the lightness (0 for black and 100 for white objects), the red-green coordinate (positive values are red, negative values are green), and the yellow-blue coordinate (yellow for positive values, blue for negative values), respectively. The samples show a slight color change over time, which is more intense with increasing silver concentration. This occurrence is connected to a marginal and non-significant rise in the a* and b* values.

### Coatings antibacterial efficiency

The results show the ability of coatings with different amounts of silver to inhibit Gram-negative microorganisms represented by *E.coli* (Table 2) and Gram-positive microorganisms such as *S.aureus* (Table 3) evaluated through the application of the ISO 22196 protocol.

**Table 2:** Antibacterial activity of Ag01 and Ag05 treated against *E.coli*

Time	T0			T1			T2			T3		
	r1	r2	r3	r1	r2	r3	r1	r2	r3	r1	r2	r3
Inoculum (CFU/ml)^**a**^	5.6 × 10^6^	5.6 × 10^6^	5.6 × 10^6^	6.0 × 10^6^	6.0 × 10^6^	6.0 × 10^6^	6.0 × 10^6^	6.0 × 10^6^	6.0 × 10^6^	6.0 × 10^6^	6.0 × 10^6^	6.0 × 10^6^
Recovery counts untreated Ag0/T0 (CFU/cm^2^)	1.4 × 10^4^	1.2 × 10^4^	1.6 × 10^4^	1.4 × 10^4^	1.2 × 10^4^	1.6 × 10^4^	1.1 × 10^4^	1.4 × 10^4^	1.4 × 10^4^	2.2 × 10^4^	2.1 × 10^4^	2.2 × 10^4^
Validity recovery counts Ag0/T0^**b**^	0.03	0.03	0.03	0.03	0.03	0.03	0.03	0.03	0.03	0.03	0.03	0.03
Recovery counts untreated Ag0/T24h^**c**^	1.2 × 10^4^	8.8 × 10^3^	1.0 × 10^4^	1.6 × 10^3^	1.6 × 10^3^	1.6 × 10^3^	3.8 × 10^3^	7.5 × 10^3^	7.5 × 10^3^	8.7 × 10^3^	9.0 × 10^3^	8.7 × 10^3^
Recovery counts treated Ag01/T24h (CFU/cm^2^)	< 1	< 1	< 1	< 1	< 1	< 1	< 1	< 1	< 1	17	16	17
Recovery counts treated Ag05/T24h (CFU/cm^2^)	< 1	< 1	< 1	< 1	< 1	< 1	< 1	< 1	< 1	< 1	< 1	< 1
Antibacterial activity (*R*)01	4.01	3.94	4.00	3.80	3.88	3.88	3.80	3.90	3.90	2.75	2.75	2.74
Antibacterial activity (*R*)05	4.01	3.94	4.00	3.80	3.88	3.88	3.80	3.90	3.90	3.95	3.95	3.94

a = range 1.0 × 10^6^ CFU/ml – 6.0 × 10^6^ CFU/ml; conditions for a valid test: b = (*L*max–*L*min)/*L*mean ≤ 0.2; c > 6.2 × 10^1^ cells/cm^2^

**Table 3:** Antibacterial activity of Ag01 and Ag05 treated against *S. aureus*

Time	T0			T1			T2			T3		
	r1	r2	r3	r1	r2	r3	r1	r2	r3	r1	r2	r3
Inoculum (CFU/ml)^**a**^	3.8 × 10^6^	3.8 × 10^6^	3.8 × 10^6^	6.0 × 10^6^	6.0 × 10^6^	6.0 × 10^6^	6.0 × 10^6^	6.0 × 10^6^	6.0 × 10^6^	6.0 × 10^6^	6.0 × 10^6^	6.0 × 10^6^
Recovery counts untreated Ag0/T0 (CFU/cm^2^)	1.1 × 10^4^	1 × 10^4^	1 × 10^4^	1.4 × 10^4^	1.3 × 10^4^	1.4 × 10^4^	1.5 × 10^4^	1.5 × 10^4^	1.5 × 10^4^	2.2 × 10^4^	2.2 × 10^4^	2.3 × 10^4^
Validity recovery counts Ag0/T0^**b**^	0.01	0.01	0.01	0.01	0.01	0.01	0.01	0.01	0.01	0.01	0.01	0.01
Recovery counts untreated Ag0/T24h^**c**^	5 × 10^3^	3.7 × 10^3^	7.5 × 10^3^	3.8 × 10^3^	7.5 × 10^3^	7.5 × 10^3^	1.7 × 10^4^	1.6 × 10^4^	1.7 × 10^4^	1.2 × 10^3^	8.7 × 10^3^	1 × 10^3^
Recovery counts treated Ag0.1/T24h (CFU/cm^2^)	< 1	< 1	< 1	< 1	< 1	< 1	< 1	< 1	< 1	8	7	8
Recovery counts treated Ag0.5/T24h (CFU/cm^2^)	< 1	< 1	< 1	< 1	< 1	< 1	< 1	< 1	< 1	< 1	< 1	< 1
Antibacterial activity (*R*)01	3.73	3.57	3.88	3.80	3.88	3.88	3.21	3.20	3.21	2.66	2.66	2.66
Antibacterial activity (*R*)05	3.73	3.57	3.88	3.80	3.88	3.88	3.21	3.20	3.21	3.56	3.56	3.56

a = range 1.0 × 10^6^ CFU/ml – 6.0 × 10^6^ CFU/ml; conditions for a valid test: b = (*L*max–*L*min)/*L*mean ≤ 0.2; c > 6.2 × 10^1^ cells/cm^2^

**Fig. 4:**
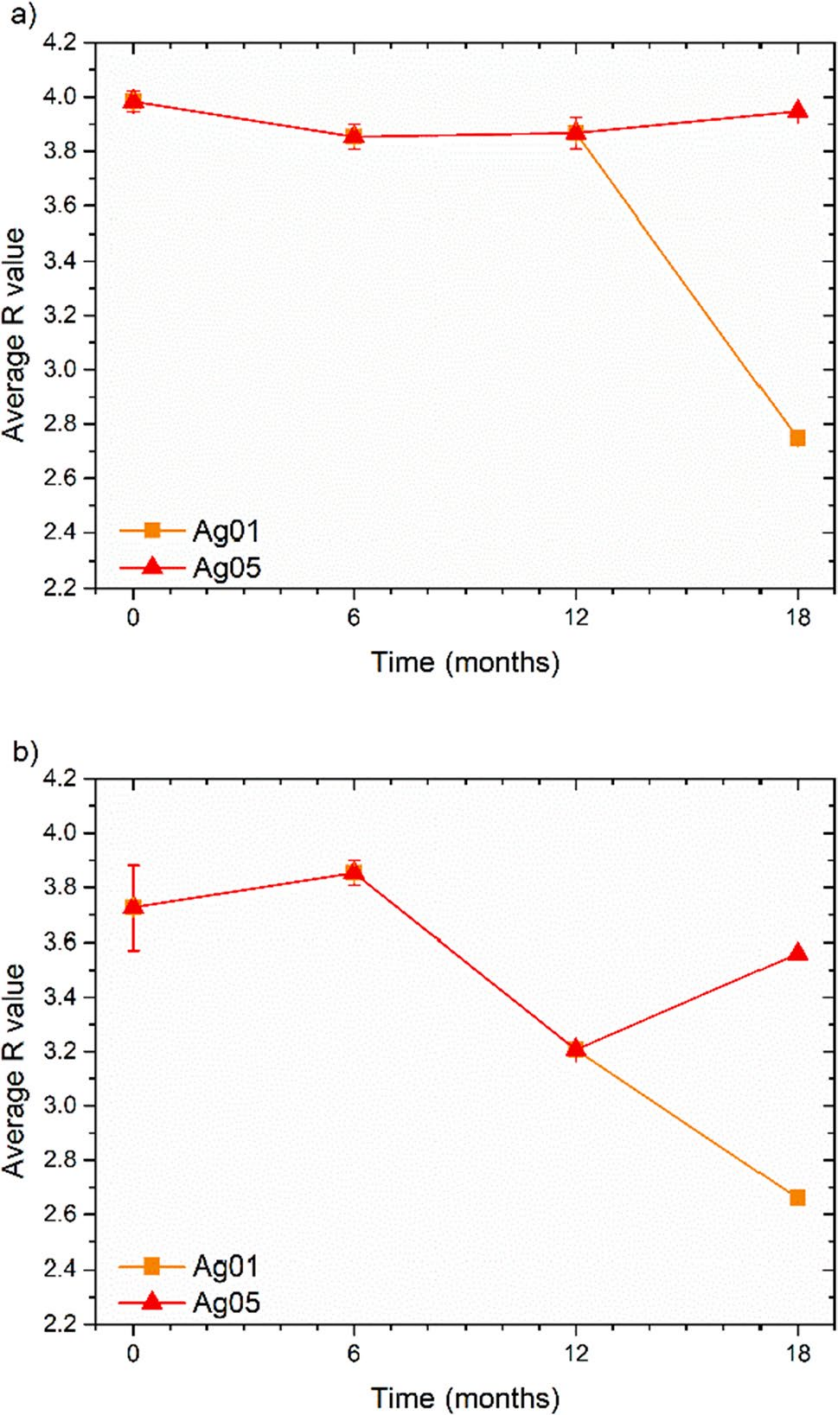
Evolution of *R* against *E.coli* (**a**) and *S.aureus* (**b**) at different time

Figure 4a and Fig. 4b represent the average antibacterial activity of sample Ag01 and Ag05 against *E.coli* and *S.aureus*, respectively. These surfaces showed protective efficacy over time, particularly when silver is present in a higher concentration (Ag 0.5) with antibacterial activity values of 3.95 and 3.56 assessed at the end of their shelf-life. It is observed that these surfaces exhibited antibacterial effects compared to the controls at each time of the 18 months-test. At time T3, corresponding to the end of the hypothetical 18-month shelf-life, an average antibacterial activity R0.1 and R0.5 of 2.75 and 3.95 for *E.coli* and 2.66 and 3.56 for *S.aureus*, respectively, was observed.

### Coatings liquid resistance

A recent work has demonstrated the good durability of these coatings, whose protective properties are not affected by the presence of silver (Calovi et al. 2023). However, the high reactivity of the silver granules towards water suggests evaluating the behavior of the coatings in contact with possible aggressive solutions. The liquid resistance test is typically employed to evaluate the impact of functional filler on the barrier capabilities of wood coatings (Calovi and Rossi 2023a, 2023b; Yan et al. 2019a, 2019b). The values of the detected color change Δ*E* are correlated with the level of discoloration displayed by the coatings when they come into contact with the particular test solutions (GB/T11186.3–90 1990) (see Table S2 in Supplementary Materials).

**Fig. 5:**
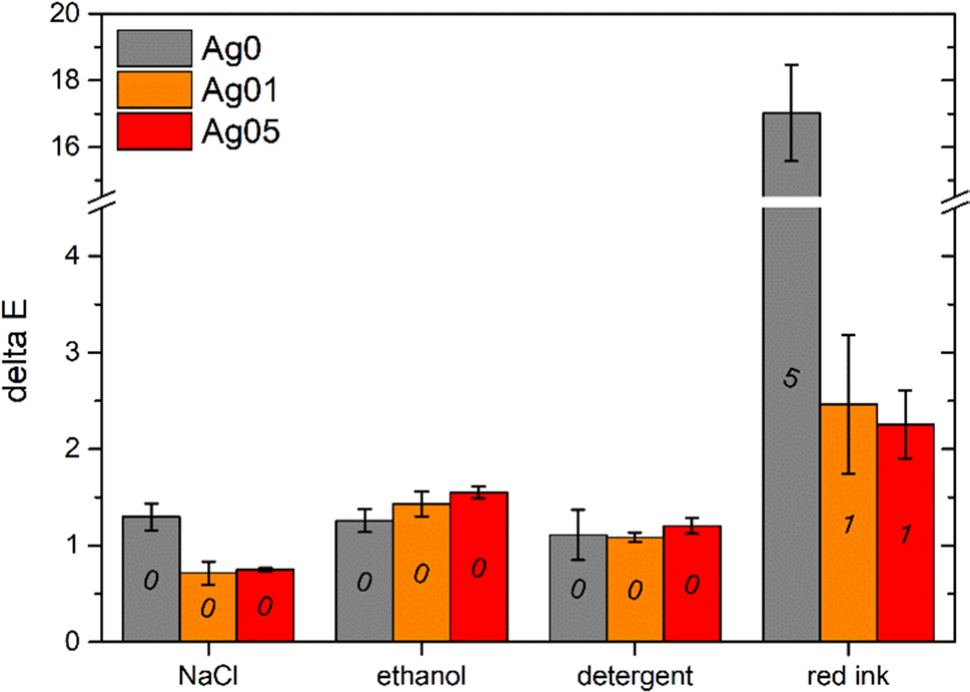
Coatings color changes after contact with liquids. The numbers above the columns are representative of the discoloration levels reported in Table 2 of Supplementary Materials

Figure 5 shows the outcomes of the chemical resistance test of the three series of coatings, along with the corresponding discoloration levels. Both the NaCl, ethanol, and detergent solutions do not cause a noticeable color change in the samples, whose discoloration levels amount to 0. The presence of silver does not influence this aspect, as the behavior of samples Ag01 and Ag05 is comparable to the outcome of coating Ag0, free of silver. Conversely, the red ink induces a less evident color change in the two samples Ag01 and Ag05 compared to the reference clear coat Ag0. As the levels of color change in the presence of silver are quite limited, it is possible to neglect the phenomena of discoloration of the coatings in contact with NaCl, ethanol, and detergent solution.

**Fig. 6:**
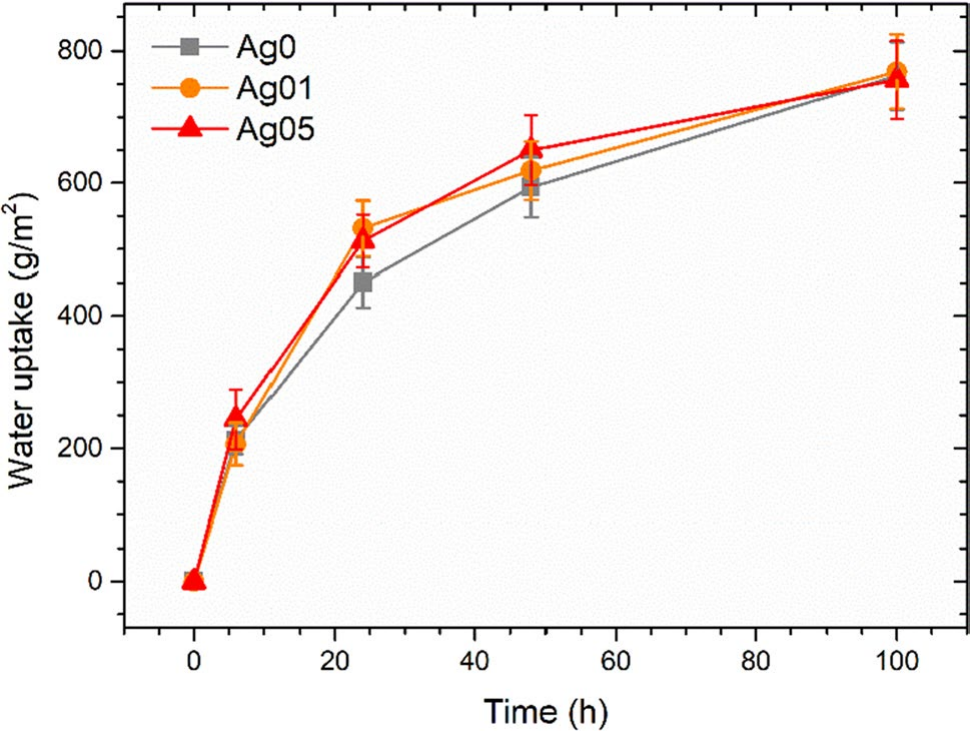
Evolution of the water uptake during the test

The liquid resistance test, however, only yields qualitative data that are associated to chromatic alterations in the coatings. Thus, the samples were further evaluated carrying out the liquid water uptake test, in order to determine the true quantitative influence of the silver additions on the barrier effect of the coatings. Figure 6 reveals the findings of the test, displaying the evolution of water uptake phenomena. The curves of the three series of coated samples are quite superimposable throughout the entire test. Silver appears to cause a slight increase in solution absorption between 24 and 48 h. However, the variability of the results suggests that the behavior of samples Ag01 and Ag05 is comparable to the outcome of coating Ag0.

**Fig. 7:**
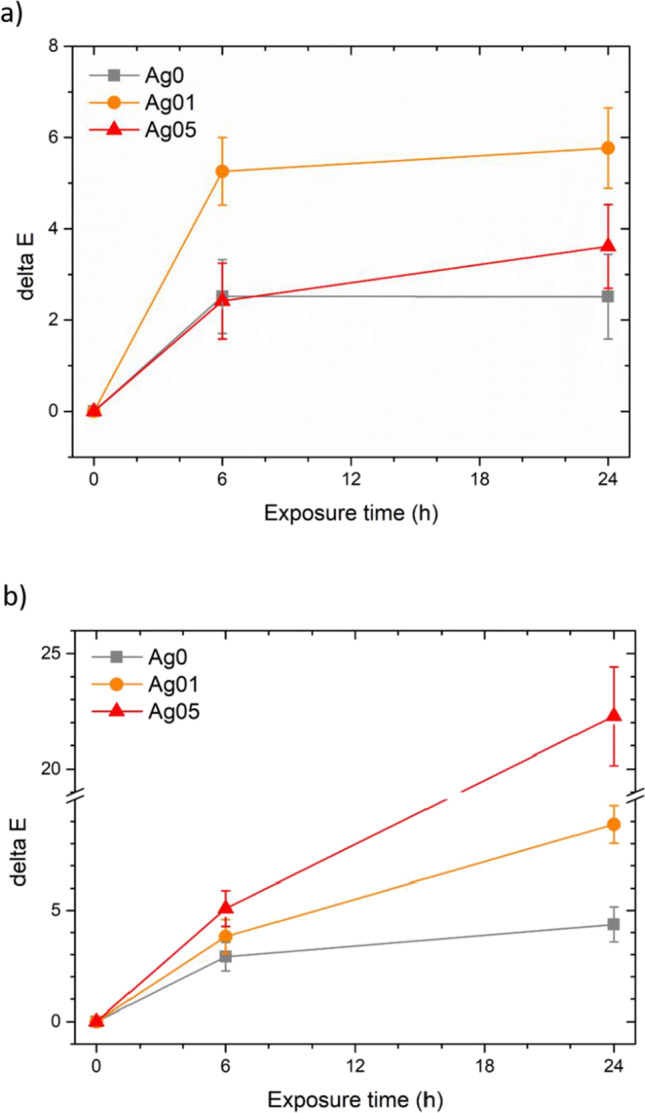
Color evolution during the resistance to perspiration test, employing water (**a**) and synthetic sweat solution (**b**)

Finally, to simulate the prolonged contact of the coatings with human skin and the continuous surface washing processes, the samples were subjected to the resistance to perspiration test. Figure 7 shows the evolution of the color change Δ*E* of the coatings in contact with the demineralized water (Fig. 7a) and the synthetic sweat solution (Fig. 7b). Both test solutions cause a non-negligible color change during the first 6 h of testing. However, while water did not further degrade the coating after 6 h, the subsequent contact with the synthetic sweat solution causes a continuous color change in the samples. This phenomenon is evidenced by the increase in silver content. In general, silver is affected by testing and results in substantial changes in the appearance of the coatings.

This outcome is highlighted by the images in Fig. S1 of Supplementary Materials, which show the evolution of the appearance of the samples during the test. The reference sample Ag0 does not undergo noticeable alterations with both test solutions. Otherwise, sample Ag01 shows similar darkening phenomena both with water and with synthetic sweat. Finally, sample Ag05 does not particularly suffer from contact with water, but exhibits effective color change caused by the synthetic sweat solution. Despite the color change of the samples, the silver content in the coatings does not change following contact with the two test solutions. In fact, the EDXS analyses show that samples Ag01 and Ag05 possess about 0.2 wt.% and 1.3 wt.% of silver, respectively, in accordance with the previous work (Calovi et al. 2023), even after 24 h in contact with water and synthetic sweat.

## Discussion

Colloidal silver confirms to be an excellent resource as a pigment for water-borne paints, thanks to its high solubility in water and its strong coloring efficiency. Furthermore, the final shade of the coating can be designed according to the concentration of colloidal silver granules, producing color layers ranging from light yellow to dark red, increasing the amount of silver. The addition of the water-borne paint by means of the Ag-containing solution does not influence the spray deposition process, as the three series of coatings have similar thicknesses and surface finishing. At the same time, silver does not cause macroscopic defects in the polyurethane layer, which appears compact and homogeneous, but it provides a vivid orange-red coloring to the coatings. Furthermore, the coatings showed good aesthetic stability over time, as shown in Fig. 3. The Δ*E* slight increases with the concentration of silver, but it still falls within a range of values so low as to be almost negligible. Indeed, taking into account that the literature establishes a threshold of Δ*E* ≥ 1 as the minimum noticeable variation visible to the human eye (Calovi et al. 2021b; Mokrzycki and Tatol 2011), the Δ*E* values of sample Ag05, which amount to 1.84 after a duration of 18 months, indicate a significant level of aesthetic stability. It is therefore possible to assert that colloidal silver introduces an intense and specific color into the coating, which however is long-lasting and constant. Unlike other antimicrobial agents (Fernandez-Saiz et al. 2009; Hong et al. 2023; Jamili et al. 2019), colloidal silver does not cause undesired color evolutions in the antibacterial layer over time. Therefore, colloidal silver performs an excellent function as an alternative pigment, whose desired antibacterial properties are associated with a better chromatic stability than typical antimicrobial agents.

Recently, the need for timely and effective pandemic plans has emerged, and one of the preventive strategies against infectious pathogens is to have surfaces with broad-spectrum antimicrobial activity with prolonged action. The presence of coatings with biocidal activity on wooden surfaces may, on the bases of the results of this study, represent a possible system for minimizing this risk. Silver is an antimicrobial that does not give rise to drug resistance mechanisms (Rudramurthy et al. 2016). As reported in numerous studies, the affinity for sulfur and phosphate groups explains the antimicrobial activity of AgNPs. Sulfur-containing proteins in the cell wall and phosphorus-containing nucleic acids would bind to them, causing damage with subsequent cell death (Franci et al. 2015).

In addition, other mechanisms of action of the antibacterial activity have been hypothesized. In this study, it has been observed how silver nanoparticles were released with silver ions each time. The binding of silver ions with the sulfur proteins of the cell wall and cytoplasmic membrane of bacteria causes an increase in permeability which leads to the cell envelope rupture. The free silver ions can deactivate respiratory enzymes and generate reactive oxygen species by interrupting adenosine triphosphate (ATP) production with intracellular damages induced by oxidative stress. Furthermore, silver ions with the sulfur and phosphorus of DNA cause problems in DNA replication and denature ribosomes with inhibition protein synthesis. In addition, silver nanoparticles affect bacterial signal transduction and tyrosine residues can lead to cell apoptosis and termination of cell multiplication (Yin et al. 2020). The differences in the results of antimicrobial activity observed against *E.coli* and *S.aureus* can be attributed, as reported in previous works, to the biological characteristics of the cell wall of Gram-positives, which is thicker than that of Gram-negatives, resulting in a peptidoglycan layer of 30–80 nm. The long-term stability of antibacterial activity confirms the performance studied previously and referred to other types of treated samples with simulated sweat resistance tests (such as synthetic sweat) and disinfection and abrasion resistance tests (such as scrub) (Calovi et al. 2021a). Finally, the results of the antibacterial activity values (*R* = 3.9 and *R* = 3.5) at the end of the shelf-life pointed out how such coatings may represent alternatives in terms of prolonged antimicrobial activity compared with the biocide agents currently in use. The emerging problem of biocide resistance and difficulties in performing constant and uniform activities associated with surface sanitization procedures could be contained and health conditions could be improved.

The liquid resistance test evidenced good performance of the silver-based filler in contact with various solutions. In fact, samples Ag01 and Ag05 do not exhibit particular discoloration phenomena, in accordance with the behavior of the pure polyurethane matrix. Furthermore, since colloidal silver imparts a reddish hue to the coating, the test performed with red ink results in a more evident color change in sample Ag0, produced with the transparent paint. The test outcome suggests that colloidal silver can be used as a pigment for wood paints, which do not undergo aesthetic alterations following cleaning processes (contact with detergent solutions or ethanol) or due to contact with saline solutions. Similarly, the silver-based filler does not alter the barrier performance of the paint, as it does not introduce structural defects into the polyurethane matrix. Consequently, the liquid water uptake test showed similar performance between the three series of samples. The overall water uptake values and trend are very low, comparable with recent literature works (Calovi and Rossi 2023a, 2023b), but also much lower than other organic coatings which revealed water uptake values that can even reach 8000 g/m^2^ (Kymäläinen et al. 2022). Finally, the resistance to perspiration test showed good compatibility of the paint with aqueous solutions, while the synthetic sweat solution produced substantial color changes in silver-containing coatings. The combination of water and temperature favored the partial oxidation of the silver, which results in a slight darkening of sample Ag01 (final Δ*E* of about 6). This phenomenon is less evident in sample Ag05 (final Δ*E* of about 4), as the starting hue is already so dark that it is challenging to appreciate a further color change as a result of the darkening of the sample. Typically, silver suffers from oxysulfidation reactions in atmosphere with high sulfide concentrations (Elechiguerra et al. 2005; Graedel 1992). However, at low sulfide concentration, silver nanoparticles can undergo a sort of oxidative dissolution/precipitation mechanism (Liu et al. 2011). Therefore, nanoscale silver oxidation processes can also occur in the absence of sulfur (Moore and Codella 1988), in normal oxygenated environments (Han et al. 2011; Oates et al. 2013; Qi et al. 2010), such as that of the test expressed in Fig. 7a. Otherwise, the reaction with the synthetic sweat solution causes marked discoloration in silver-containing coatings (Fig. 7b). The phenomenon is accentuated according to the concentration of filler, as it is associated with silver oxidation processes. In fact, silver experiences oxidation activity promoted by acidic environments (Mittelman et al. 2013; Zhang et al. 2011). The tarnishing process is accompanied by darkening of the silver (Burleigh et al. 2001; Singh et al. 1983): consequently, the higher the concentration of the filler, the darker the coating appears following contact with the acid solution. Therefore, the coatings show good resistance to detergent solutions and to water, but silver particularly suffers from prolonged contact with acid solutions. This aspect warns against the application of colloidal silver in wood paints for applications in public environments: repetitive contact with human skin could cause the chromatic alterations observed in the samples in Fig. S1 (Supplementary Materials).

However, such low quantities of silver-based fillers ensure against possible toxicological phenomena due to the degradation of the coating and the release of silver (Bouwmeester et al. 2009; Klaine et al. 2008; Lansdown 2010; Reidy et al. 2013). It is well established that silver poisoning (argyria) can occur through both oral (ingestion) (Chang et al. 2006; Johnston et al. 2010) and dermal (skin) (Trop et al. 2006) exposure. Most of the risk assessments associated with silver focus on the development of argyria. The World Health Organization (WHO) has established a no observable adverse effect level (NOAEL) of 6.5-µg/kg body weight per day (bw/day) (Proykova 2014), indicating the maximum amount of silver that can be consumed daily without adverse effects. Similarly, the US Environmental Protection Agency (US EPA) has provided a reference dose (RfD) of 5 µg/kg bw/day for chronic oral silver exposure (EPA 1991). For a 70-kg adult, this translates to a total daily intake of 350 µg of silver. On the other hand, the European Chemicals Agency (ECHA) has implemented even more stringent limits, setting the daily intake at 1.2 µg/kg, which corresponds to 84 µg of silver for a 70-kg adult (Proykova 2014). Nonetheless, taking into account the minimal quantity of silver incorporated into the surface and assuming that the interaction between the human hand and the coating takes place on an area of approximately 120 cm^2^, the samples could release such a negligible amount of silver that it would mostly remain well within the more stringent limits set by the US Environmental Protection Agency (US EPA) and the European Chemicals Agency (ECHA). In fact, considering the values imposed by the ECHA, equal to 84 µg per day and a contact area of 120 cm^2^ (average size of a hand), the maximum release allowed would be 0.7 µg/cm^2^ of silver per day. However, since the paint deposition yield is about 10 µg/cm^2^, sample Ag05 contains about 0.05 µg/cm^2^ of silver. Therefore, even if the coating released the entire silver content within it in a single day, this would fall within the limits set by the reference agencies.

In conclusion, colloidal silver serves as a fascinating versatile dye in wood coatings. It possesses the advantage of not inducing noticeable flaws in the polyurethane layer, which appears consistent and uniform. On the other side, it imparts a vibrant shade of orange-red to the coatings, constant over time. Additionally, this research emphasizes the enduring antibacterial effectiveness of these coatings, suggesting their potential as alternatives with extended antimicrobial properties compared to the currently employed biocidal agents. By addressing the growing concern of biocide resistance and the challenges of consistent and uniform surface sanitization, these coatings have the potential to enhance health conditions and mitigate associated difficulties. The coatings also demonstrated favorable resilience to detergent solutions and water, although prolonged exposure to acid solutions can have a detrimental effect on silver. This observation cautions against the use of colloidal silver in wood paints for public settings, as repeated contact with human skin could result in coatings’ changes in color. Nevertheless, despite the potential degradation effects caused by contact with humans, the small quantity of silver incorporated into the coatings provides reassurance regarding the minimal cytotoxicity of the product.

## Supplementary Information

Below is the link to the electronic supplementary material.

Supplementary file1 (PDF 564 KB)

## Data Availability

All data generated or analyzed during this study are included in this published article.
