# Supplementary material for: Antibacterial efficiency over time and barrier properties of wood coatings with colloidal silver
Source: Appl Microbiol Biotechnol. 2023 Aug 8;107(19):5975–86. doi: 10.1007/s00253-023-12710-1 (PMC10484803; doi:10.1007/s00253-023-12710-1)
Supplement: Supplementary file 1 — Supplementary file1 (PDF 564 KB) [file 253_2023_12710_MOESM1_ESM.pdf]

Journal: Applied Microbiology and Biotechnology

Manuscript Title: Antibacterial efficiency over time and barrier properties of wood coatings with colloidal silver

Authors: Massimo Calovi[1*], Valentina Coroneo[2], Stefano Rossi[1]

1Department of Industrial Engineering, University of Trento, Via Sommarive 9, 38123 Trento Italy;

2 Department of Medical Sciences and Public Health, University of Cagliari, S.P.8 Monserrato, 09042 Cagliari, Italy;

*Corresponding author: massimo.calovi@unitn.it Tel.: +39-0461-282403

Table S1 Materials for the antibacterial test conditions

Antibacterial surfaces	Wood + Colloidal silver in polyurethane matrix - 2.5 cm × 2.5 cm
Control surface	Wood + polyurethane matrix - 2.5 cm × 2.5 cm
Cover film	Polypropylene film - 2.0 cm × 2.0 cm
Bacterial strains	*Escherichia coli* ATCC 25922 and *Staphylococcus aureus* ATCC 6538
Volume of inoculum	100 µl

Table S2 Color change values corresponding to the level of discoloration

Level	Degree of Discoloration	Color Difference
0	no color change	≤1.5
1	very slight discoloration	1.6-3.0
2	slight color change	3.1-6.0
3	apparent discoloration	6.1-9.0
4	severe color change	9.1-12.0
5	complete discoloration	>12.0

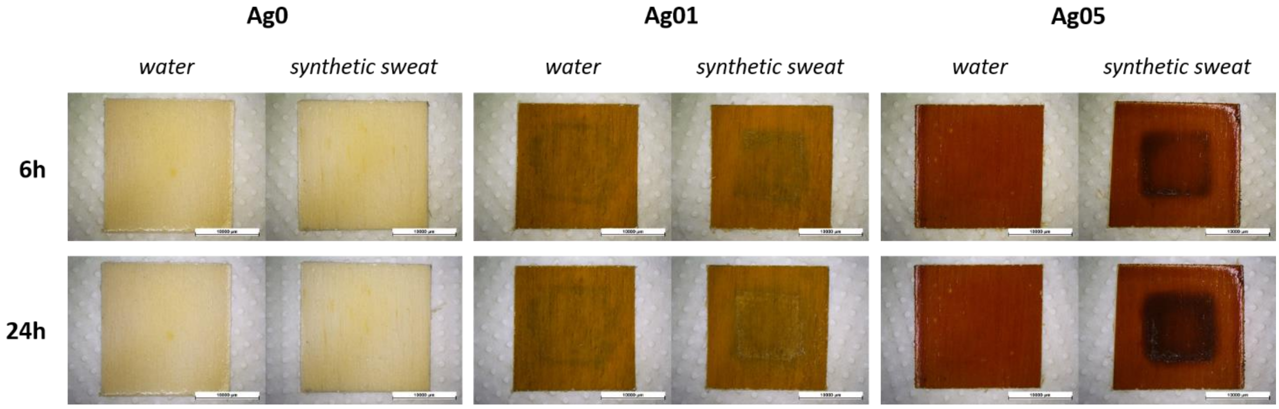

Fig. S1 Evolution of the aspect of the samples during the resistance to perspiration test
